# Comparative Developmental Transcriptomics Reveals Rewiring of a Highly Conserved Gene Regulatory Network during a Major Life History Switch in the Sea Urchin Genus *Heliocidaris*

**DOI:** 10.1371/journal.pbio.1002391

**Published:** 2016-03-04

**Authors:** Jennifer W. Israel, Megan L. Martik, Maria Byrne, Elizabeth C. Raff, Rudolf A. Raff, David R. McClay, Gregory A. Wray

**Affiliations:** 1 Department of Biology, Duke University, Durham, North Carolina, United States of America; 2 Schools of Medical and Biological Sciences, The University of Sydney, Sydney, New South Wales, Australia; 3 Department of Biology, Indiana University, Bloomington, Indiana, United States of America; 4 Center for Genomic and Computational Biology, Duke University, Durham, North Carolina, United States of America; University of Bath, UNITED KINGDOM

## Abstract

The ecologically significant shift in developmental strategy from planktotrophic (feeding) to lecithotrophic (nonfeeding) development in the sea urchin genus *Heliocidaris* is one of the most comprehensively studied life history transitions in any animal. Although the evolution of lecithotrophy involved substantial changes to larval development and morphology, it is not known to what extent changes in gene expression underlie the developmental differences between species, nor do we understand how these changes evolved within the context of the well-defined gene regulatory network (GRN) underlying sea urchin development. To address these questions, we used RNA-seq to measure expression dynamics across development in three species: the lecithotroph *Heliocidaris erythrogramma*, the closely related planktotroph *H*. *tuberculata*, and an outgroup planktotroph *Lytechinus variegatus*. Using well-established statistical methods, we developed a novel framework for identifying, quantifying, and polarizing evolutionary changes in gene expression profiles across the transcriptome and within the GRN. We found that major changes in gene expression profiles were more numerous during the evolution of lecithotrophy than during the persistence of planktotrophy, and that genes with derived expression profiles in the lecithotroph displayed specific characteristics as a group that are consistent with the dramatically altered developmental program in this species. Compared to the transcriptome, changes in gene expression profiles within the GRN were even more pronounced in the lecithotroph. We found evidence for conservation and likely divergence of particular GRN regulatory interactions in the lecithotroph, as well as significant changes in the expression of genes with known roles in larval skeletogenesis. We further use coexpression analysis to identify genes of unknown function that may contribute to both conserved and derived developmental traits between species. Collectively, our results indicate that distinct evolutionary processes operate on gene expression during periods of life history conservation and periods of life history divergence, and that this contrast is even more pronounced within the GRN than across the transcriptome as a whole.

## Abstract 2

The ecologically significant life history transition in sea urchins from feeding to yolk-dependent larval stage was accompanied by major evolutionary changes in gene expression during development.

## Author Summary

An important challenge in both evolutionary and developmental biology is understanding the genetic and molecular basis for the differences in phenotype that we observe between species. Although changes to regulatory interactions during development often play a critical role in this process, we lack detailed examples of how these differences evolve within the context of developmental gene regulatory networks (GRNs) and across the broader transcriptome as a whole. To better understand how evolutionary changes in gene regulation contribute to phenotypic differences between species, we developed a novel analytical framework to compare gene expression profiles between sea urchin species that exhibit either planktotrophic (feeding) or lecithotrophic (nonfeeding, yolk-dependent) development. We found that the shift from planktotrophy to lecithotrophy was accompanied by substantial changes to underlying gene expression profiles, and that this contrast was even more pronounced within the well-defined developmental GRN in sea urchins than across the transcriptome as a whole. Our analyses also identify cases of expression change in key regulatory genes with known developmental roles and further highlight novel candidates that may underlie derived traits in the lecithotrophic species. The comparative framework that we develop and model here is a valuable tool for identifying evolutionary changes in gene expression between species and can be readily implemented in other systems.

## Introduction

Changes in regulatory gene interactions during development play a major role in the evolution of phenotypic differences between species. Yet how these regulatory changes are positioned, facilitated, and constrained within the context of broader gene regulatory networks (GRNs) remains a largely unresolved question for both evolutionary and developmental biologists.

The GRN underlying development of sea urchins is the most comprehensive and well-studied GRN of any animal to date [1–20] and provides a valuable framework to investigate how changes in gene expression during development contribute to phenotypic evolution between species [21–25]. This network contains over 200 experimentally verified regulatory interactions and covers major aspects of early development in sea urchins, from the unfertilized egg through the formation of a planktonic feeding larva [1–20]. Decades of comparative genetic and biochemical research have shown that the GRN exhibits a high level of conservation among sea urchin species, including *Strongylocentrotus purpuratus*, *Paracentrotus lividus*, and *L*. *variegatus*, which are separated by more than 50 million years (Myr) [26,27]. Furthermore, key features of the sea urchin GRN are present even in distantly related echinoderms [21,23]. This raises questions about the evolvability of regulatory interactions within the network, particularly among sea urchins: is the network highly constrained and therefore evolutionarily inflexible? Or is the network malleable and simply optimized to produce a stereotypic planktonic feeding larva and thus maintained by stabilizing selection?

To explore these questions, we investigated the sea urchin GRN in the context of a major life history transition that involved extensive and dramatic changes in developmental mechanisms: the switch from feeding (planktotrophic) to nonfeeding (lecithotrophic) larval development [28]. Although planktotrophy is the ancestral mode of development in sea urchins [28,29], lecithotrophy has independently evolved at least 14 times in this clade [30,31]. Lecithotrophs produce fewer, larger eggs that are rich in maternal proteins and lipid stores, allowing these species to develop through metamorphosis without feeding [32,33]. The evolution of lecithotrophy is nearly always accompanied by an extensive suite of derived developmental features, including altered cleavage geometry, reduction or loss of key morphological features of the larval support system (e.g., gut, skeleton, and ciliated band), greatly accelerated development of the imaginal juvenile rudiment, and much earlier metamorphosis [28–30,34]. Here, we use “lecithotrophy” to refer to this syndrome of coderived features that characterize the life history transition [35].

We focused on the genus *Heliocidaris*, which encompasses one of the most comprehensively studied life history transformations from a developmental perspective in any animal [32,36–48]. A wealth of research comparing development in *H*. *tuberculata*, a species with a planktotrophic larva, and *H*. *erythrogramma*, a species with a lecithotrophic larva, has contributed to our understanding of the dramatic changes in developmental mechanisms that accompanied the rapid transition from planktotrophic to lecithotrophic development in this genus (divergence time = 5 Myr) [37,39,41–45,47]. While some developmental processes are delayed in *H*. *erythrogramma* relative to the planktotrophs (e.g., formation of the larval skeleton), others are accelerated (e.g., patterning of the juvenile body plan) [36,48]. In particular, key patterning mechanisms such as dorsoventral axis specification, the establishment of the primary signaling center of the embryo, and early cell fate specification differ between the two species. Notably, some of these modifications involve developmental mechanisms that were previously conserved for 10s–100s of millions of years before changing dramatically and rapidly during the evolution of lecithotrophy in *H*. *erythrogramma* [38–40,49].

An important goal for this study was to identify evolutionary changes in developmental gene expression that might have contributed to this dramatic shift in life history strategy within *Heliocidaris*. While many changes in gene expression are likely the simple result of an overall accelerated development and have nothing to do with causing the life history transition, others may involve altered regulatory interactions (i.e., network “rewiring”) that produced changes in level, timing, spatial extent, or even complete loss of gene expression in the lecithotroph. Results from previous studies suggest that several GRN components operate at a different time during development or interact with different genes in *Heliocidaris* [41,43,45,47].

To move beyond a case-by-case approach and detect evolutionary changes in gene expression throughout the transcriptome during the evolution of lecithotrophy, we developed a comparative clustering strategy that identifies statistically supported differences in the shape of expression profiles during development, as opposed to focusing on differences at individual time points. Importantly, this approach differentiates simple cases of minor change in the level or timing of expression from more complex cases. A key aspect of this method is use of an explicit phylogenetic framework with an outgroup planktotrophic species, *L*. *variegatus*, which allowed us to polarize differences in gene expression to specific branches of the phylogeny. Using this comparative clustering strategy, we contrasted genome-wide expression dynamics across development, from an unfertilized egg to an early larva, in *H*. *tuberculata*, *H*. *erythrogramma*, and *L*. *variegatus*. We also examined evolutionary changes in gene expression within the context of the well-defined sea urchin GRN and further leveraged our comparisons to identify novel players likely involved in sea urchin development.

Our results reveal that changes in gene expression profiles were more numerous during the evolution of lecithotrophy than during the persistence of planktotrophy, and that this contrast is even stronger within the GRN. We found evidence for both conservation and divergence of GRN linkages in *H*. *erythrogramma*, as well as significant changes in the expression of genes with known roles in patterning the larval skeleton, which is delayed and highly reduced in this species. Collectively, our results indicate that the transition from planktotrophy to lecithotrophy involved extensive changes to the expression of genes underlying key developmental processes.

## Results

**Fig 1:**
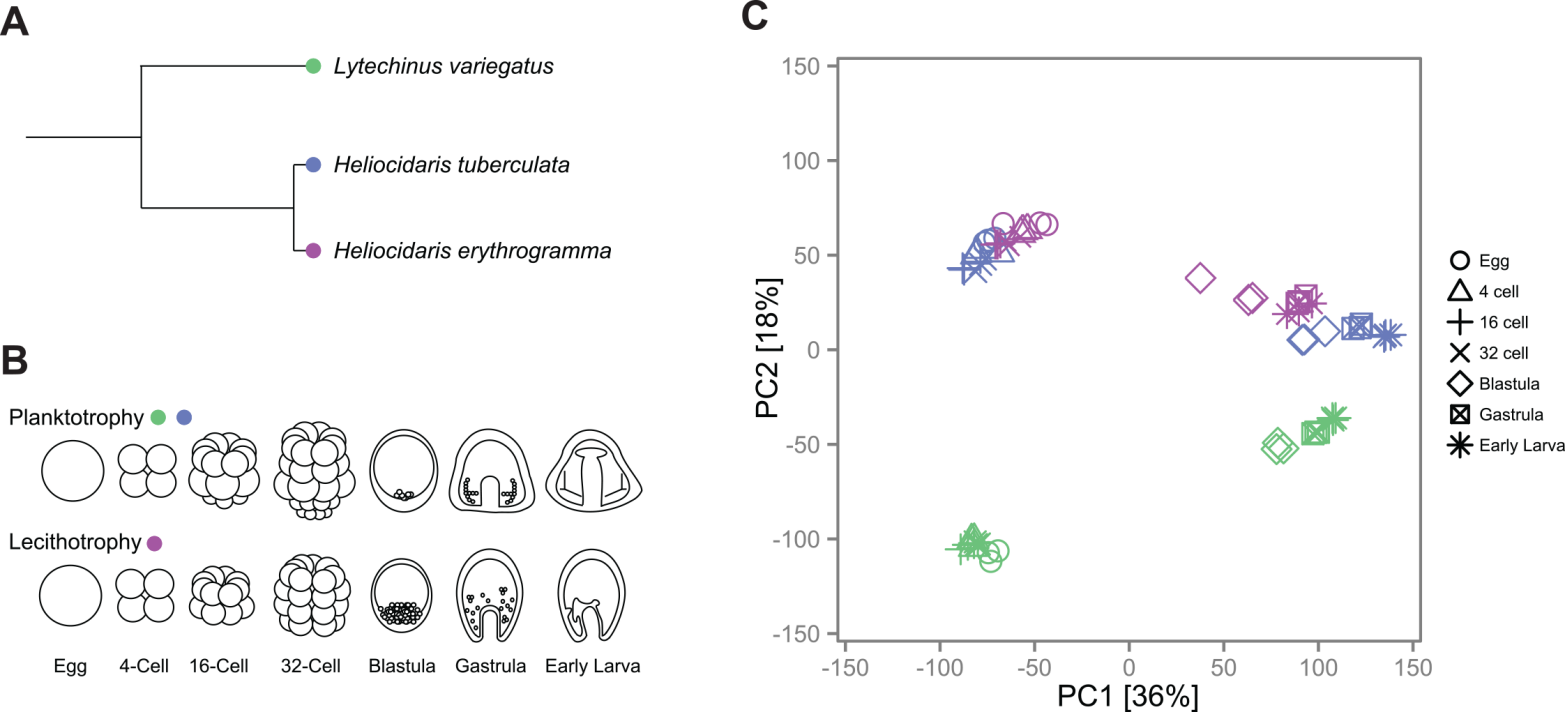
Gene expression during sea urchin development reflects known phylogenetic relationships between species. (**A**) Our study includes three sea urchin species: the sister species *H*. *tuberculata* (planktotroph) and *H*. *erythrogramma* (lecithotroph), which diverged approximately 5 Myr, and an outgroup species *L*. *variegatus* (planktotroph), which diverged approximately 35–45 Myr ago [44, 50]. (**B**) Our developmental time course includes seven stages across each species, from unfertilized eggs to early larvae. (**C**) Principal component (PC) analysis of gene expression (S1 Data). PC1 explains 36% of the overall variation and clearly separated early (egg through 32-cell stage) from later developmental stages (blastula through early larva), whereas PC2 separated *L*. *variegatus* from the two *Heliocidaris* species, corresponding to their phylogenetic relationships.

In order to identify evolutionary changes in gene expression underlying lecithotrophy in *Heliocidaris*, we used Illumina RNA-seq to measure expression dynamics across early development in three sea urchin species: the lecithotroph *H*. *erythrogramma*, the closely related planktotroph *H*. *tuberculata* (divergence time = 5 Myr) [44], and an outgroup planktotroph *L*. *variegatus* (divergence time = 35–45 Myr) (Fig 1A) [50]. We sampled seven stages of development in triplicate for each species, from unfertilized eggs to early larvae (Fig 1B). These data form the basis for the analyses described below.

### Global Gene Expression Patterns during Development Recapitulate Phylogeny

Given the striking differences between planktotrophic and lecithotrophic development, we were interested in the extent to which variation in global gene expression patterns was explained by life history strategy, as opposed to phylogenetic relationships between species. To address this question, we performed a principal component analysis (PCA) to identify the major sources of variance in our transcriptome dataset. The first principal component (PC) explained 36% of the variation and distinguished early developmental stages (egg through 32-cell stage) from later ones (blastula through early larva) in all three species (Fig 1C). This separation corresponds roughly to maternal versus zygotic gene expression profiles. PC2, which explained 18% of the variance, separated *L*. *variegatus* from the two *Heliocidaris* species, corresponding to their phylogenetic relationships. PC3, which explained 13% of the variation, further distinguished *H*. *erythrogramma* from *H*. *tuberculata*, separating by life history strategy (S1 Fig). These results identify developmental stage as the main source of differences in global gene expression profiles among the samples we examined. In contrast, phylogeny and life history strategy were relatively minor contributors to expression divergence in the transcriptome as a whole.

In order to gain further insight into the extent of gene expression change between species, we computed the transcriptome divergence (1 − ρ, Spearman’s correlation coefficient) between each species pair at individual stages within our time course (S2 Fig). Consistent with our PC analysis, our results reflect the known phylogenetic relationships between species. The divergence of both *Heliocidaris* species, relative to the outgroup *L*. *variegatus*, is highest in the egg and tends to decrease across development. Early stages (egg through 32-cell) were more divergent than later ones (blastula through early larva), again likely reflecting maternal versus zygotic gene expression profiles. Our results also show that *H*. *erythrogramma* is more divergent with respect to the outgroup than *H*. *tuberculata* across development, except at the gastrula stage.

### Changes in Gene Expression Profiles Were More Numerous during the Evolution of Lecithotrophy Than during the Persistence of Planktotrophy

**Fig 2:**
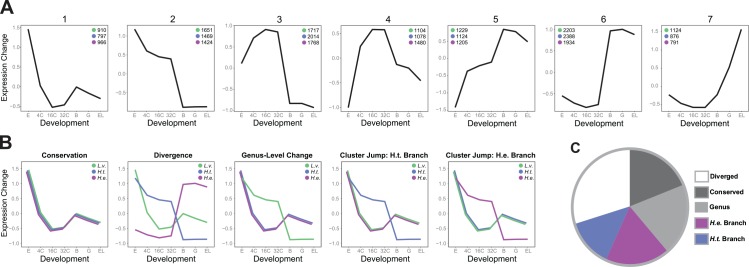
Comparative clustering strategy reveals cases of expression profile conservation and change within a phylogenetic framework. **(A)** Cluster centroids identified in the outgroup *L*. *variegatus* are shown (S2 Data), along with the number of genes in each species that were assigned to a given cluster. Green = *L*. *variegatus*, blue = *H*. *tuberculata*, and purple = *H*. *erythrogramma*. The *x*-axis represents developmental time, and the *y*-axis represents expression change. **(B)** Comparative clustering strategy identifies genes that exhibit the same expression profile among all three species (conservation), differ among all three species (divergence), differ between *L*. *variegatus* and the *Heliocidaris* species (genus-level change), or change specifically along a particular branch of the phylogeny (cluster jump in *H*. *tuberculata* or *H*. *erythrogramma*). **(C)** Using a comparative clustering strategy (see 2B and Methods), we characterized expression profile divergence (white, 29.8%), conservation (dark grey, 19.1%), and genus-level change (light grey, 19.8%) across the transcriptome. We also detected branch-specific change in *H*. *tuberculata* (blue, 13.5%) and *H*. *erythrogramma* (purple, 17.8%) (S1 Table).

An important goal for this study is to identify changes in gene expression that may have contributed to the evolution of lecithotrophy. Because development is a dynamic, time-dependent process, we sought a method to identify statistically supported evolutionary differences in the shapes of gene expression profiles during development. We implemented a comparative clustering strategy to detect substantial differences in expression profiles across development regardless of absolute expression level and within an explicit phylogenetic framework (see Methods for details). First, we used fuzzy c-means clustering to group similar expression profiles in our outgroup species *L*. *variegatus* into seven clusters, representing distinct phases of expression during development (Fig 2A). Next, we separately assigned *H*. *tuberculata* and *H*. *erythrogramma* expression profiles to these clusters based on their association with *L*. *variegatus* cluster centroids. Within each species, we also designated a separate cluster for genes with very low expression (VLE) across the time course, which we further refer to as the “very low-expressed” cluster. This strategy allowed us to easily identify which genes exhibited the same expression profile among all three species (conservation), differed among all three species (divergence), or changed specifically along a particular branch of the phylogeny, which we refer to as “cluster jumps” (Fig 2B).

By these criteria, more genes show conserved expression profiles between *H*. *tuberculata* and *H*. *erythrogramma* than between *L*. *variegatus* and either of the *Heliocidaris* species (Fig 2C). This result shows that overall divergence in embryonic gene expression follows phylogenetic relationships rather than developmental strategy, reinforcing our PCA (Fig 1C). Unlike a PCA, however, this approach allowed us to polarize evolutionary differences in expression. We observed more cluster jumps along the *H*. *erythrogramma* branch than the *H*. *tuberculata* branch (Fisher’s exact test, adjusted *p* = 1.06 x 10^−17^), and the ratio of expression change between the *Heliocidaris* branches (~1.3x) remained similar regardless of the initial number of clusters formed (S2 Table). This result suggests that, although most expression differences appear to accumulate neutrally, a subset is related to the evolution of a derived developmental mode in *H*. *erythrogramma*.

On the *H*. *tuberculata* and *H*. *erythrogramma* branches, jumps occurred between every combination of clusters, which led us to ask whether there were any significant differences with respect to the nature of gene expression changes during the evolution of lecithotrophy. We found that significantly more genes jumped from cluster 3 to cluster 4 in *H*. *erythrogramma* compared to *H*. *tuberculata* (Fisher’s exact test, adjusted *p* = 1.23 x 10^−06^). A significantly higher number of genes also jumped from cluster 6 and cluster 7 into the VLE group in *H*. *erythrogramma* (Fisher’s exact test, adjusted *p* = 8.98 x 10^−07^ and adjusted *p* = 1.72 x 10^−10^, respectively). These results indicate that although many different types of gene expression change occurred during the evolution of lecithotrophy, the derived developmental mode in *H*. *erythrogramma* is primarily distinguished by both subtle shifts and loss of expression.

**Fig 3:**
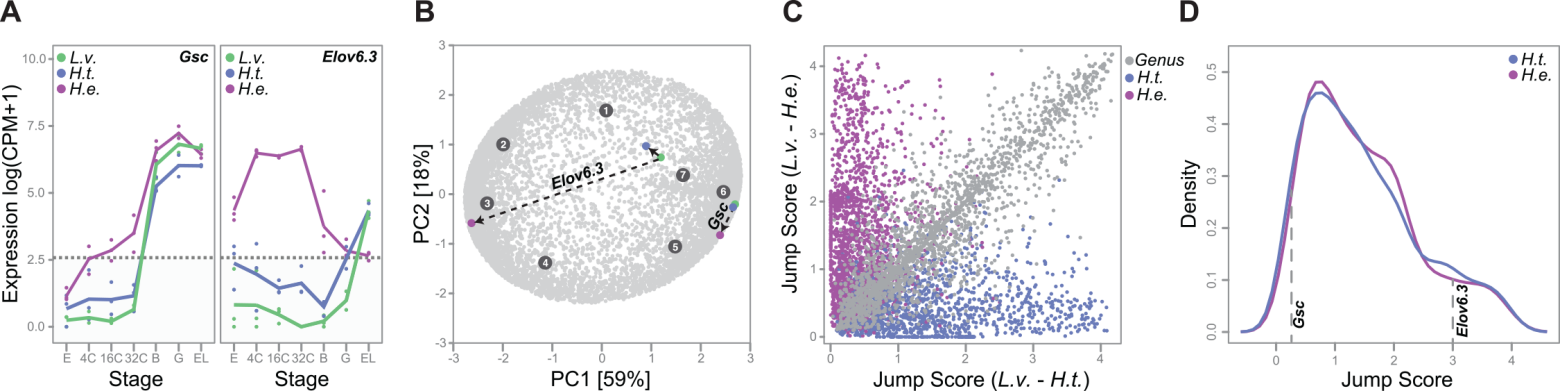
Subtle shifts are more common than dramatic changes in gene expression between species. **(A)** Along the *H*. *erythrogramma* branch, *Gsc* displays a subtle change in expression and is slightly accelerated compared to the planktotrophs. Accordingly, *Gsc* is assigned to cluster 6 in the planktotrophs and cluster 5 in *H*. *erythrogramma*. In contrast, *Elov6*.*3* exhibits a dramatic change in expression and is assigned to cluster 7 in the planktotrophs and cluster 3 in the lecithotroph. Biological replicates are represented as circles, and average expression profiles across replicates are represented as lines. Expression values below the horizontal line are less than 5 counts per million (cpm) and are designated as VLE (S1 Data). **(B)** In the PCA plot, each ortholog is a light grey circle and each cluster centroid is a dark grey circle. The PCA was performed on S3 Data (see Methods). To calculate a jump score from the *L*. *variegatus* ortholog of *Elov6*.*3* (green circle) to the *H*. *erythrogramma* ortholog of *Elov6*.*3* (purple circle), we calculated a scaled distance, or “jump score,” from the first two PC loadings (see Methods). This calculation was repeated to obtain the distance between the *L*. *variegatus* and the *H*. *tuberculata* ortholog (blue circle). The position of *Gsc* orthologs for each species is also shown. All profiles designated as VLE have the coordinates (0,0). **(C)** Distribution of jump scores calculated from *L*. *variegatus* to *H*. *tuberculata* (*x*-axis) and from *L*. *variegatus* to *H*. *erythrogramma* (*y*-axis) (S4 Data). Jump scores are colored according to comparative clustering classification: *H*. *erythrogramma* branch jumps are purple, *H*. *tuberculata* branch jumps are blue, and genus level changes are grey. For clarity, jump score distributions of conserved and diverged genes are plotted separately in S3 Fig. **(D)** Kernel density curves of jump scores for genes that exhibited a cluster jump in either *H*. *tuberculata* (blue) or *H*. *erythrogramma* (purple) (S4 Data).

We next sought a method to distinguish subtle from dramatic shifts in expression. For example, in *H*. *erythrogramma*, the transcription factor *Gsc* exhibited a slight acceleration in expression compared to the planktotrophs (i.e., *Gsc* jumps from cluster 6 into cluster 5 in *H*. *erythrogramma*) (Fig 3A). This acceleration in *Gsc* expression was observed previously and is consistent with an altered development of ectoderm in the lecithotroph [45]. In contrast, *Elov6*.*3*, which encodes a fatty acid elongase, exhibited a more dramatic jump in expression along the *H*. *erythrogramma* branch, moving from cluster 7 into cluster 3 (Fig 3A). This acceleration of *Elov6*.*3* expression in *H*. *erythrogramma* is consistent with the dramatically elevated lipid content of the lecithotroph egg [32,33].

To quantitatively differentiate subtle from dramatic shifts in expression, we performed a PCA of expression profiles (see Methods). For each gene, we then calculated scaled distance measures, or “jump scores,” from the *L*. *variegatus* ortholog to both the *H*. *erythrogramma* and *H*. *tuberculata* orthologs using the first two PC loadings (Fig 3B). The relationship between these jump scores and the evolutionary classifications from our prior cluster analysis is intuitive: genes that exhibited a cluster jump along the lecithotroph branch tend to have a high jump score between *L*. *variegatus* and *H*. *erythrogramma*, but a low jump score between *L*. *variegatus* and *H*. *tuberculata* (Fig 3C, purple circles). In contrast, genes that exhibited a cluster jump along the *H*. *tuberculata* branch tend to have a low jump score between *L*. *variegatus* and *H*. *erythrogramma*, but a high jump score between *L*. *variegatus* and *H*. *tuberculata* (Fig 3C, blue circles). Further, genes that were conserved between all three species tend to exhibit low jump scores in both contrasts, whereas genes that diverged between species do not display a clear pattern (S3 Fig).

We next plotted density distributions of jump scores for genes that changed along either *Heliocidaris* branch (Fig 3D). From these plots, it is evident that jumps between similar clusters are more frequent than jumps between highly distinct clusters in both species. This result indicates that while subtle divergence in gene expression profiles is frequent between species, dramatic changes are less common.

### Changes in Gene Expression during the Evolution of Lecithotrophy Are Qualitatively Distinct from Those during the Persistence of Planktotrophy

To explore whether the evolutionary conservation and differences we observed in developmental expression profiles were reflected in higher-level functional classes of genes, we performed a categorical enrichment analysis using a hypergeometric test and the GO (Gene Ontology) Biological Process ontology database (see Methods). In the divergence gene set (profile assigned to a different cluster in each species), we found significant enrichment of one metabolic category (bile acid biosynthetic process) when controlling for a false discovery rate (FDR) of 10% (S3 Table). In contrast, the conserved gene set (profile in the same cluster in all three species) was enriched by 237 categories (10% FDR) encompassing a wide range of biological processes, including many related to development (e.g., cell fate determination) (S3 Table). We did not observe any significantly enriched categories for genes that changed specifically along the *H*. *erythrogramma* or *H*. *tuberculata* branch, nor for genes that differed between *L*. *variegatus* and the *Heliocidaris* species.

Due to the large number of significantly enriched categories for our conservation gene set, we performed a parallel analysis in which we reduced the gene set background from all three-way orthologs to only those that exhibited a branch-specific change in expression (i.e., diverged and conserved genes were excluded). Similar to our earlier analysis, we did not observe any significantly enriched categories for genes that changed specifically along the *H*. *tuberculata* branch or for genes that differed between *L*. *variegatus* and the *Heliocidaris* species. However, for genes that changed specifically along the *H*. *erythrogramma* branch, we found significant enrichment of four categories when controlling for an FDR of 10%, all of which were related to developmental processes (i.e., cartilage condensation, neuron migration, midbrain development, and negative regulation of neuron differentiation) (S3 Table).

Several of the genes in these developmental categories have critical regulatory roles during embryonic or larval development in sea urchins (e.g., *dlx*, *gsc*, *myoD*, *foxC*, *pax2/5/8*). That said, some are not known to be involved in the specific developmental processes indicated by the GO category name per se in sea urchins, as these associations are based on studies in distantly related model organisms. What is clear from our results is that major changes in the expression profiles of developmental regulatory genes occurred during the evolution of lecithotrophy in *Heliocidaris*.

### Changes in Expression Differ between the GRN and the Transcriptome As a Whole

To further explore gene expression changes associated with the evolution of lecithotrophic development, we examined cluster jumps within the context of the well-established GRN of planktotrophic sea urchins. The network is broadly divided into three embryonic territories that execute distinct specification and differentiation programs for the skeletogenic, endomesodermal, and ectodermal cell lineages [1]. Both the topology and function of the GRN is highly conserved, even among distantly related echinoderm species [21,23,27]. Yet the dramatically altered skeleton, nonfunctional gut, and reorganized ectoderm of *H*. *erythrogramma* larvae suggest that changes may have occurred in the GRN during the relatively recent and rapid evolution of lecithotrophic development [36,42,46,49]. This led us to consider the following questions: to what extent have expression changes evolved in the GRN of *H*. *erythrogramma*? Are these changes characterized by heterochronic shifts in expression, and/or is the expression of particular genes lost? Are these changes localized to particular territories or specific subcircuits of those territories (e.g., specification, patterning, differentiation)? And importantly, do these expression changes likely have downstream phenotypic consequences for development?

**Fig 4:**
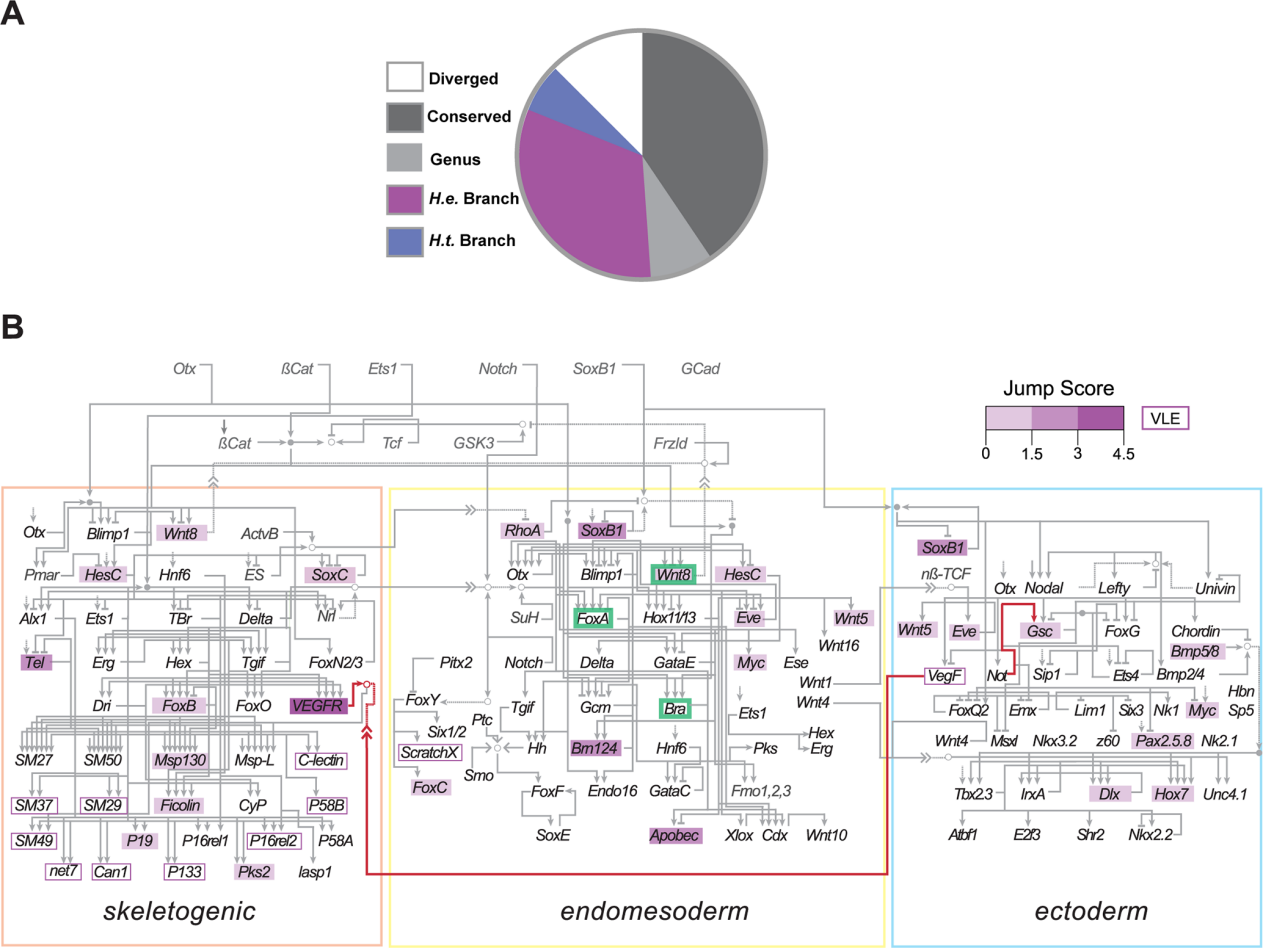
Life history strategy is a major component of gene expression divergence across the GRN. **(A)** Compared to the transcriptome, the GRN exhibits increased expression profile conservation among species (dark grey, 41.5%) and reduced divergence (white, 11.3%). Genus level and *H*. *tuberculata*-specific cluster jumps are also reduced (light grey, 8.5% and blue, 5.7%, respectively), while cluster jumps specifically along the *H*. *erythrogramma* branch increased at the GRN level compared to the transcriptome (purple, 33%) (S4 Table). **(B)** *H*. *erythrogramma*-specific branch jumps (purple) occur in each GRN territory, though they are concentrated in the skeletogenic lineage (Binomial test, *p* < 0.01). Names of genes measured in this study are shown in black, other genes in grey. The associated jump score for each *H*. *erythrogramma*-specific branch jump is represented by box color. Jumps into the VLE group are represented by open boxes. Examples of GRN change in *H*. *erythrogramma* are highlighted, including EM subcircuit compression (green boxes, S4A Fig) and altered regulatory interactions between *VegF* and *VEGFR* and between *Not* and *Gsc* (red lines, S4D–S4F Fig). Generalized categories of GRN change are depicted in S5 Fig.

In contrast to cluster jumps throughout the transcriptome (Fig 2C), we observed more conservation and less divergence of gene expression profiles within the GRN (Fig 4A). Notably, more network genes had conserved expression profiles between the planktotrophs than between the two *Heliocidaris* species, suggesting that within the GRN life history strategy, and not phylogenetic position, is the principal determinant of interspecies differences in gene expression. Consistent with this result, we observed a much higher ratio of expression change between *H*. *erythrogramma* and *H*. *tuberculata* within the GRN compared to the transcriptome as a whole (branch length ratio ~5.8x, Fig 4A). Our results also reveal that changes along the *H*. *erythrogramma* branch occurred in all three principal territories of the GRN: skeletogenic, endomesoderm (EM), and ectoderm (Fig 4B, S4 Table). Several of these changes in gene expression suggest evolutionary “rewiring” of GRN interactions during the rapid evolution of lecithotrophic development in *Heliocidaris* (see Discussion).

### Distinct Patterns of Evolutionary Change in Gene Expression among Embryonic Territories

Changes on the *H*. *erythrogramma* branch occurred at all levels of the GRN, from genes involved in early fate specification to those involved in terminal differentiation of larval cells. A striking example of early change is the delayed expression of *Wnt8*, one of the first zygotically activated genes in the GRN (S4A Fig). This delay was observed previously by Kauffman and Raff using northern blots [43]. In planktotrophs, *Wnt8* is initially expressed in the micromeres, which arise asymmetrically at 4th cleavage and constitute the major signaling center of the embryo [51]. Wnt8-mediated signaling initiates a series of inductive interactions along the animal–vegetal axis that specify the major cell fates within the EM. Inhibition of Wnt8 signaling in species with planktotrophic larvae causes extensive endomesodermal defects [51]. Unlike planktotrophs, *H*. *erythrogramma* has an equal 4th cleavage, does not produce micromeres, and endomesodermal fate specification is delayed [37,39]. The delayed expression of *Wnt8* is likely related to these changes in fate specification. Nonetheless, *Wnt8* expression remains necessary for EM specification in *H*. *erythrogramma* [43]. Furthermore, our results show that components of EM specification downstream of Wnt8 signaling in planktotrophs, including *Bra* and *FoxA* exhibit conserved expression profiles between *L*. *variegatus* and the *Heliocidaris* species (S4A Fig). These results suggest that the EM specification subcircuit has been conserved in function but compressed in timing during the evolution of lecithotrophic development in *Heliocidaris*.

Following specification, the EM territory separates into distinct endodermal and nonskeletogenic mesodermal (NSM) lineages, with endoderm contributing to the larval gut and the NSM lineage contributing to blastocoelar cells, pigment cells, muscle cells, and coelomic pouch cells of the larva [52–55]. The larva of *H*. *erythrogramma* has a small, nonfunctional gut that lacks the mouth, tripartite gastrointestinal tract, and associated muscles present in planktotrophic larvae [56]. In *H*. *erythrogramma*, we observed that the expression profile of the GRN gene *Abopec*, which encodes a differentiation product of the larval gut, fails to rise during gastrulation as it does in planktotrophs (S4B Fig). In addition, although another endodermal differentiation gene *Endo16* had a conserved expression profile between all three species, its level of expression in *H*. *erythrogramma* was substantially reduced compared to the planktotrophs (S4B Fig). These evolutionary changes in gene expression are likely related to the development of the reduced, nonfuctional larval gut in *H*. *erythrogramma*. We also observed altered expression of the regulatory gene *Brn1/2/4* in *H*. *erythrogramma* (S4B Fig). In planktotrophic larvae, *Brn1/2/4* encodes a transcription factor that is expressed in both the gut and ectoderm [57]. The lower level of expression may play a role in the dramatically altered development of one or both of these territories in *H*. *erythrogramma*.

Prior to metamorphosis in planktotrophs, the left coelomic pouch (comprised of NSM and primordial germ cells) enlarges and signals to the overlying ectoderm to initiate patterning of the juvenile body plan [58,59]. This interaction usually occurs weeks after fertilization in planktotrophic species. In *H*. *erythrogramma*, the development of the left coelomic pouch is greatly accelerated, and adult body plan formation is initiated just after gastrulation, less than 2 d after fertilization [48,56]. Our results provide evidence of both conservation and divergence within the coelomic pouch NSM subcircuit of the GRN. In planktotrophs, the transcription factor *FoxY*, which is located at the top of the regulatory hierarchy underlying coelomic pouch cell specification [15], is conserved in expression between all three species but exhibits an elevated level of expression in *H*. *erythrogramma* (S4C Fig). Other conserved coelomic pouch genes include *FoxF* and *SoxE*. However, expression of *FoxC*, a downstream target of *FoxY*, is accelerated and expression of *ScratchX*, another *FoxY* target, is lost in *H*. *erythrogramma* (S4C Fig) [15]. These observations suggest that both compression and rewiring of the coelomic pouch subcircuit have contributed to the greatly accelerated development of the adult body plan in *H*. *erythrogramma*.

While expression changes along the *H*. *erythrogramma* branch occurred in all three territories of the GRN, cluster jumps were concentrated within the skeletogenic lineage (Binomial test, *p* < 0.01). Both planktotrophs and lecithotrophs specify skeletogenic cells, but in *H*. *erythrogramma*, these cells do not migrate and assemble into the stereotypical ring pattern of planktotrophic larvae and produce a much smaller and simpler skeleton [36]. Therefore, it was not surprising that VEGF/VEGFR signaling, which provides guidance cues and differentiation signals to migratory skeletogenic cells in planktotrophs [60], appears to be dramatically altered in *H*. *erythrogramma*: expression of the VEGF ligand, which signals to the migrating skeletogenic cells from the overlying ectoderm, is greatly reduced and expression of its receptor VEGFR is greatly modified (S4D Fig). The expression of several downstream effector genes involved in the biomineralization and construction of the larval skeleton [61–63] was also lost during the evolution of lecithotrophic development in *Heliocidaris*, including *SM-49* and *C-lectin* (S4E Fig). However, the expression of other skeletogenic genes was maintained at a reduced level in *H*. *erythrogramma* (e.g., *SM-50* and *P58-a*). These genes may play a conserved role in larval skeletogenesis or may be necessary for formation of the juvenile skeleton, which is greatly accelerated in *H*. *erythrogramma* [56].

Lastly, we examined gene expression changes within the ectodermal territory. In planktotrophs, distinct oral and aboral territories of gene expression are established within the ectoderm, separated by the ciliary band [55]. In *H*. *erythrogramma*, the ectoderm is substantially reorganized, including loss of an oral–aboral distinction and gain of novel expression domains [42,64]. Although differences between the planktotrophs and *H*. *erythrogramma* were not as dramatic in the ectoderm as in other territories, we nonetheless observed several expression changes consistent with the evolution of a modified ectoderm in *H*. *erythrogramma*. As noted previously, the ectoderm of *H*. *erythrogramma* no longer expresses the ligand VEGF, an important factor involved in patterning the larval skeleton, at an appreciable level. Similarly, the transcription factor *Pax2/5/8*, which is also involved in skeletal patterning [16,65], is delayed and reduced in *H*. *erythrogramma* (S4F Fig). We also observed delayed expression of the transcription factors *Dlx* and *Hox7* (S4F Fig). Both *Dlx* and *Hox7* are involved in differentiation of aboral ectoderm in planktotrophic larvae [13], and previous studies suggest important roles for these genes in formation of the juvenile body plan [66,67]. Further, a particularly striking example of change within the ectodermal subcircuit is the earlier activation of *Gsc* than its upstream regulatory *Not* in *H*. *erythrogramma* (S4F Fig). This altered regulatory interaction is consistent with the loss of oral–aboral distinction in the lecithotroph.

### Coexpression Analysis Identifies Novel Candidate Genes for Gut and Neural Development

Because the genetic basis for the evolution of lecithotrophy is unlikely to reside only in genes that are part of the GRN, we next explored whether we could leverage our knowledge of developmental differences between *H*. *erythrogramma* and the planktotrophs, along with our comparative transcriptomic dataset, to identify novel candidate genes for future empirical studies.

**Fig 5:**
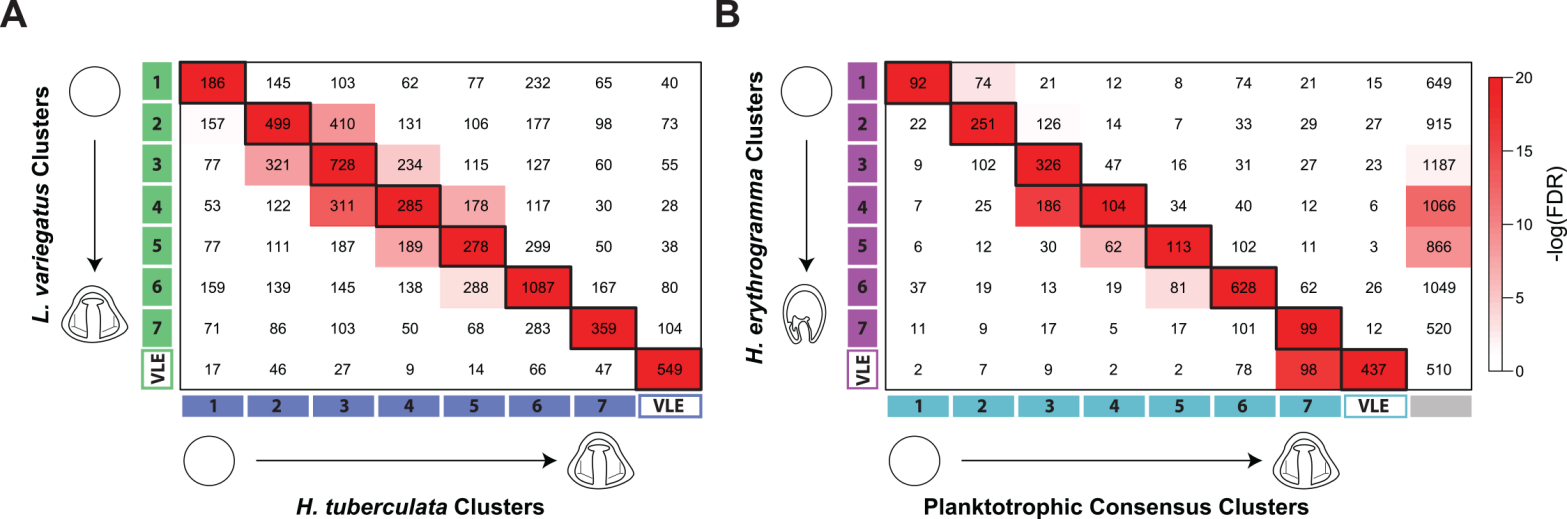
Genes coexpressed during planktotrophic development significantly overlap with those coexpressed during lecithotrophic development. **(A)** Overlap matrix of *H*. *tuberculata* and *L*. *variegatus* cluster assignments (S1 Table). The *x*-axis represents *H*. *tuberculata* clusters, and the *y*-axis represents *L*. *variegatus* clusters. Each cell contains the number of intersecting genes, and the cell color represents the significance of the intersection. Cells along the matrix diagonal are “consensus clusters” between the planktotrophic species (e.g., consensus cluster 1 contains 186 genes that are coexpressed in both *H*. *tuberculata* cluster 1 and *L*. *variegatus* cluster 1). Consensus cluster cells are outlined in black. In general, genes coexpressed below the matrix diagonal represent genes that have accelerated expression profiles in *H*. *tuberculata* compared to *L*. *variegatus*, whereas genes coexpressed above the matrix diagonal represent genes that have delayed expression in *H*. *tuberculata* compared to *L*. *variegatus*. **(B)** Overlap matrix of planktotrophic consensus cluster and *H*. *erythrogramma* cluster assignments (S1 Table). The *x*-axis represents planktotrophic consensus clusters (detailed in **A**), except for the grey cluster, which represents genes that did not exhibit consensus expression profiles between planktotrophs (i.e., genes that were off-diagonal in **A**). The *y*-axis represents *H*. *erythrogramma* clusters. Each cell contains the number of intersecting genes, and the cell color represents the significance of the intersection. Cells along the matrix diagonal are coexpressed between all three species and are outlined in black. In general, genes coexpressed below the matrix diagonal represent genes that have accelerated expression profiles in the planktotrophs compared to *H*. *erythrogramma*, whereas genes coexpressed above the matrix diagonal represent genes that have delayed expression in the planktotrophs compared to *H*. *erythrogramma*.

First, we re-examined the results from our comparative clustering analysis of gene expression profiles across the transcriptome (Fig 2A). However, instead of treating genes as isolated units, we considered them as members of coexpression groups. This allowed us to explore the movement of groups of coexpressed genes, as opposed to individual cluster jumps, between species. We first created an overlap matrix of *L*. *variegatus* and *H*. *tuberculata* cluster membership in order to visualize the genes in common between each planktotroph cluster pair (Fig 5A). Cluster pairs with significant overlap between the planktotrophs (Fisher’s exact test, adjusted *p* < 0.05) primarily fall along the matrix diagonal, indicating that genes coexpressed early in *L*. *variegatus* development significantly overlap with those coexpressed early in *H*. *tuberculata* development. The same is true for genes coexpressed during later developmental stages. In general, genes coexpressed below the matrix diagonal represent genes that have accelerated expression profiles in *H*. *tuberculata* compared to *L*. *variegatus*, whereas genes coexpressed above the matrix diagonal represent genes that have delayed expression in *H*. *tuberculata* compared to *L*. *variegatus*. We further refer to the eight coexpressed groups specifically along the matrix diagonal as “consensus clusters” (e.g., consensus cluster 1 contains 186 genes that are coexpressed in both *H*. *tuberculata* cluster 1 and *L*. *variegatus* cluster 1).

Next, we examined the overlap of these planktotroph consensus clusters with *H*. *erythrogramma* clusters (Fig 5B). Similar to our previous analysis, cluster pairs with significant overlap between *H*. *erythrogramma* and the planktotrophs (Fisher’s exact test, adjusted *p* < 0.05) primarily fall along the matrix diagonal. However, significant overlap of cluster pairs off-diagonal is also present, including the overlap of consensus cluster 7 and the VLE cluster of *H*. *erythrogramma*, which contains 98 genes.

**Fig 6:**
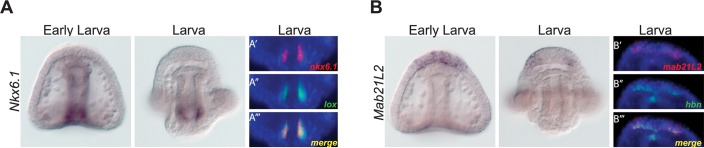
Cross-species transcriptome comparison reveals novel candidates for planktotrophic development. **(A)** Whole mount in situ hybridization reveals expression of *Nkx6*.*1* is restricted to the endoderm in the early larva and to the hindgut in the larva in *L*. *variegatus*. (A’–A”‘) Double fluorescent in situ hybridization shows that *Nkx6*.*1* and hindgut marker *Lox* colocalize in the hindgut of *L*. *variegatus* larvae. **(B)** Whole mount in situ hybridization reveals expression of *Mab21l2* is first restricted to the apical plate and later to two lateral patches in the anterior neurogenic ectoderm in *L*. *variegatus* larvae. (B’–B”‘) Double fluorescent in situ hybridization reveals that *Mab21l2* and anterior neurogenic ectoderm marker *Hbn* colocalize in the anterior neurogenic ectoderm of *L*. *variegatus* larvae.

The planktotroph/*H*. *erythrogramma* overlap matrix (Fig 5B) provides a valuable framework to query the movement of coexpression groups between species in order to identify promising candidates for future investigation. From our comparative analysis of genes within the GRN, it was clear that many expression changes within *H*. *erythrogramma* occur in a predictable manner. For example, *H*. *erythrogramma* does not develop a functional larval gut or skeleton, and the expression of genes that underlie gut and skeletal differentiation in planktotrophs is often reduced or lost in *H*. *erythrogramma*. Therefore, we hypothesized that novel candidates for genes involved in gut and skeletal differentiation in planktotrophs would also be reduced or lost during the evolution of lecithotrophy. To explore this possibility, we examined the overlap of the planktotroph consensus cluster 7 with the VLE cluster in *H*. *erythrogramma*, which contains 98 genes (Fig 5B). Of these genes, several are currently in the GRN or have known roles in the development of differentiated skeleton (e.g., *Otp*) or gut (e.g., *Hb9*), tissues that are highly conserved in planktotrophs but not in *H*. *erythrogramma* [36,56]. We chose to further investigate the transcription factor *Nkx6*.*1*, whose expression has been observed in the developing gut of other organisms [68,69]. Our results show that in *L*. *variegatus*, *Nkx6*.*1* expression is restricted to the hindgut of the larva and colocalizes with known hindgut marker *Lox* by double fluorescent in situ hybridization (Fig 6A) [70]. This result suggests a role for *Nkx6*.*1* in planktotrophic gut differentiation and demonstrates the utility of our comparative approach as a tool to identify novel candidate genes for expression changes underlying specific derived phenotypes in *H*. *erythrogramma*.

To further investigate the usefulness of our comparative approach as a gene discovery tool, we examined the group of 628 genes coexpressed between all three species in cluster 6. We hypothesized that these genes are likely involved in developmental processes shared by both planktotrophs and the lecithotroph. Not surprisingly, many GRN genes are assigned to this group, including a large portion of the ectodermal subcircuit. We chose to focus on *Mab21l2*, which is involved in nervous system development in other organisms [71,72]. In the *L*. *variegatus* larva, *Mab21l2* expression is first restricted to the apical plate and later to two lateral patches in the anterior neurogenic ectoderm (Fig 6B). Further, *Mab21l2* colocalizes with *Hbn*, a known marker of anterior neurogenic ectoderm by double fluorescent in situ hybridization [73]. The anterior neurogenic ectoderm later gives rise to serotonergic neurons, which develop as bilaterally symmetric, apical ganglia in all three species [74,75]. Together, these results suggest that *Mab21l2* may play a conserved role in anterior neurogenic ectoderm development in sea urchins.

### Coexpression Analysis Reveals Candidate Genes for Lecithotrophic Development

In addition to using the planktotroph/*H*. *erythrogramma* overlap matrix to identify novel candidate genes for planktotrophic development, we also used the matrix to highlight changes that may be important for the evolution of lecithotrophic-specific traits in *H*. *erythrogramma*. Specifically, we examined genes that are coexpressed above the matrix diagonal, as these generally represent genes that have accelerated expression profiles in *H*. *erythrogramma* compared to both planktotrophs. We then sorted this set of genes into two broad categories: (1) genes that are expressed in all three species, but accelerated specifically in *H*. *erythrogramma* (e.g., the GABA transporter *Gat3*, the methyl sterol oxidase *Sc4mol*.*1*, and the olfactory G-protein *Golf*) (S6A Fig) and (2) genes that are VLE in the planktotrophs but not in *H*. *erythrogramma* (e.g., the sensory GPCR *Opn5L*, the calcium binding protein *Rgn*, and the transcription factor *Hox9/10*) (S6B Fig.). Of note, these genes provide additional candidates for experimental verification independent of the known GRN components, and they further illustrate the potential of whole transcriptome comparisons to identify novel candidates independent of prior information.

## Discussion

Life history is critical to every organism’s interaction with its environment, yet we know far less about the genetic and developmental basis for the evolution of life history than we do for morphology and physiology. This is in part because the major developmental and genetic model organisms are not closely related to species with divergent life histories [76] and in part because life history transitions often involve changes in complex suites of functionally interrelated traits that make them challenging to study [77]. We devised an approach based on comparative developmental transcriptomics to gain insights into the evolution of complex phenotypic changes and applied it to the life history switch from planktotrophy to lecithotrophy within the sea urchin genus *Heliocidaris*. This approach can be applied to nonmodel organisms and further benefits from, but is not dependent upon, information about gene functions and interactions during development.

### Towards an Analytical Framework for Comparative Developmental Transcriptomics

A significant challenge in evolutionary genetics is distinguishing gene expression differences relevant to a trait of interest from overall divergence in gene expression. Because the evolution of lecithotrophy in *Heliocidaris* involves dramatic changes to key developmental processes [32,33,36–48], we hypothesized that the underlying molecular mechanisms likely include major changes in the expression profiles of developmental regulatory genes. We therefore established an unsupervised analytical framework to identify cases where a gene expression profile is conserved among species with the ancestral life history but divergent in the species with the derived life history.

Importantly, our approach considers expression profiles, rather than individual time points, for orthologous genes throughout the transcriptome. Using expression profiles as the unit of analysis is critical for developmental data because development is an inherently time-dependent process. We then use an outgroup in the context of a well-established phylogeny to infer the polarity of differences in these expression profiles. While a handful of previous studies have introduced methodology to compare gene expression profiles between pairs of species across embryonic development, they were not able to polarize expression differences due to the absence of an outgroup [78–81]. A few other studies have included multiple species but focused exclusively on identifying conserved expression profiles, rather than branch-specific changes [82–85]. Our approach draws on elements of these previous studies to quantify divergence in the overall expression profile of each gene in a branch-specific manner and independent of expression level.

Notably, this approach can distinguish subtle shifts in timing from more extensive changes in expression (Fig 3), providing clues about conservation and change in gene function. A relatively small shift in the timing of gene expression (heterochrony), for instance, implies a conserved function that simply occurs at a different time during development (e.g., *Gsc*, Fig 3A). A more substantial change in expression, such as a gain of maternal expression and early zygotic expression (e.g., *Elov6*.*3*, Fig 3A), implies a change in function during development, such as a transcription factor regulating a different developmental process or a substantial change in metabolism. Although changes in expression profiles alone cannot demonstrate a connection to a particular developmental mechanism or to a higher-level trait, this analytical framework can highlight candidate genes whose functional role can then be validated and characterized through experimental studies.

### The Evolution of Lecithotrophy Altered Natural Selection Throughout the Transcriptome

Quantifying and polarizing changes in gene expression profiles can also provide insights into the evolutionary processes that shape transcriptional regulation. Our results reveal that evolutionary divergence in embryonic gene expression across the transcriptome is dominated by the effects of phylogenetic distance. This is evident from PC analysis of individual time points and from comparative clustering analysis of expression profiles across the transcriptome. Previous studies, including some that examined development, have also found that divergence in gene expression on the transcriptome scale is positively correlated with phylogenetic distance [82,84,86]. This pattern is consistent with a time-dependent neutral process: drift fixes many eQTL (expression quantitative trait loci) of small effect size, resulting in gradual divergence in expression profiles over time [87,88].

Despite this overall time dependence in expression divergence, we also found evidence that natural selection may operate differently on the transcriptome during the transition from planktotrophy to lecithotrophy. One manifestation is a modest increase in the number of expression profile changes on the *H*. *erythrogramma* branch compared to the *H*. *tuberculata* branch, hinting at the operation of a non-neutral process. Genes with derived expression profiles in the lecithotroph show specific characteristics as a group that reinforce this interpretation: subtle shifts in expression and greatly reduced expression are both significantly enriched on the *H*. *erythrogramma* branch relative to the *H*. *tuberculata* branch. Further, the genes whose expression changed in the lecithotroph are significantly enriched for gene ontology categories related to development. The first class of derived gene expression profiles may be related to dramatic changes in the timing of key developmental processes, such as a delay in the specification and differentiation of the skeletogenic cell lineage [36]. Similarly, the second class of derived gene expression profiles may be related to the loss of other key developmental processes in the lecithotroph, such as the absence of a functional gut [56]. Together, these results suggest that the evolution of gene expression throughout the transcriptome differs during periods of conservation in life history (as in *H*. *tuberculata*) and major changes in life history (as in *H*. *erythrogramma*): the former is dominated by neutral processes (i.e., developmental systems drift) [89], while the latter adds a layer of positive selection for specific kinds of changes in the shape of expression profiles.

### The Expression of GRN Genes Evolves Quite Differently from the Transcriptome As a Whole

These patterns of gene expression evolution were even more pronounced when we only considered genes that encode known components of the developmental GRN. Along branches where life history remains conserved, gene expression changes were much less common across the GRN compared to the transcriptome as a whole. This result is consistent with a recent study demonstrating that GRN genes exhibit significant conservation of temporal activation and relative expression dynamics between distantly related planktotrophic urchin species [27]. These findings are compatible with stronger stabilizing selection on the expression of developmental regulatory genes relative to the transcriptome as a whole and fit the expectation that temporal deployment of regulatory genes during development must be precise in order for them to function correctly within a GRN.

Given this high level of GRN conservation across distantly related species, the extensive network change we observed on the *H*. *erythrogramma* branch is remarkable (Fig 4A). Further, the number of expression profile changes along the *H*. *erythrogramma* branch compared to the *H*. *tuberculata* branch was far more pronounced across the network than across the transcriptome as a whole (~5.8X compared to ~1.3X).

Taken together, results at the scales of the transcriptome and the GRN suggest a model for the evolution of gene expression profiles during the switch from planktotrophy to lecithotrophy in *Heliocidaris*. When life history is conserved, most evolutionary changes in gene expression profiles are subtle and accumulate as species diverge. Most are likely fixed through drift, with negative and stabilizing selection eliminating many large changes in gene expression. During these extended intervals, the expression profiles of developmental regulatory genes are particularly refractory to change, in keeping with the conservation of the critical developmental mechanisms they regulate. In contrast, dramatic changes in gene expression profiles become substantially more common during the evolution of massive modifications to early development and to life history. This is particularly evident among developmental regulatory genes, a large fraction of which show major changes.

### Changes in the Expression of GRN Genes Likely Affect Many Aspects of Lecithotrophic Development

Several evolutionary changes in complex phenotypes are well understood from a developmental perspective, including pigmentation patterns in flies [90–92], adaptation to a cave environment by fish [93–96], and the transition of sticklebacks from salt to freshwater habitats [97–99]. The evolution of lecithotrophy differs from these cases in that it involves extensive changes to embryonic development. Hypotheses regarding the developmental basis for the evolution of lecithotrophy emphasize the importance of increased maternal provisioning of the egg and decreased time to metamorphosis [29,30,33–35]. In principle, both modifications could be accomplished by a small number of changes in gene expression: up-regulation of yolk protein synthesis and lipid storage machinery during oogenesis and a quicker pace of otherwise unmodified GRN interactions during embryogenesis.

Examining our results from the perspective of how genes within the GRN carry out their function suggests that this simple model does not hold for the evolution of lecithotrophy in *Heliocidaris*. We observed derived gene expression profiles in *H*. *erythrogramma* throughout early development, from the earliest steps in fate specification (e.g., *Wnt8*) to terminal differentiation of larval cells (e.g., *SM-49*). These evolutionary changes took place in genes that operate broadly as well as those that operate solely within specific territories and cell lineages. Finally, the major changes in expression profiles included direct regulators of gene expression (transcription factors and cofactors) as well as signaling system components (ligands and receptors).

Transcriptome comparisons alone cannot identify the cause of these expression differences, such as whether they result from mutations in *cis* or *trans* or both. In certain cases, however, the changes suggest altered interactions among GRN components, or evolutionary “rewiring” of the network. One criterion involves cases where prior work has shown that an upstream regulator is both necessary and sufficient to activate transcription of a downstream target. If one observes the downstream gene transcribed prior to the upstream gene, then regulatory interactions must have changed. An example from our data is earlier activation of *Gsc* than its upstream regulator *Not* in *H*. *erythrogramma* (S4F Fig). A second criterion involves cases where prior work has shown that two components interact at particular time. If one observes that the partners are no longer expressed at the same time, then the interaction likely no longer occurs. An example from our data is the loss of coordinate expression of *Vegf* and *VegfR* in *H*. *erythrogramma* (S4D Fig), a key ligand-receptor interaction in planktotrophs [60]. Although the molecular basis for the change remains unknown, in both cases evolutionary rewiring seems likely.

### Evolutionary Rewiring within the Skeletogenic Cell Lineage

Focusing on gene expression changes within a specific cell lineage can provide insights into the evolution of the organismal traits they influence. The skeletogenic cell lineage is one of the first to be specified and committed in planktotrophs and later constructs the biomineral skeleton of the larva. This cell lineage shows many changes in *H*. *erythrogramma*, including delayed specification and terminal differentiation as well as reduction in the size and complexity of the resulting larval skeleton [36,37,39,100]. We observed major changes in the expression profiles of several genes related to skeletogenic cell fate specification, morphogenesis, and biomineralization, but the majority involve genes that encode terminal differentiation products (Fig 4B). This observation is consistent with two compatible interpretations. One is that the expression profiles of genes within differentiation batteries are evolutionarily less constrained because they do not have direct downstream targets. The other is that these peripheral genes encode the effector molecules that directly influence ecologically relevant traits, making them targets of positive selection for the derived life history mode.

Changes in the expression of *FoxB*, *C-lectin*, and *Msp130* in *H*. *erythrogramma* are of particular interest, as our previous study of the planktotroph *S*. *purpuratus* showed that population-level variation in the expression of these genes is correlated with variation in the size and shape of the larval skeleton [101]. Together, these results suggest that the terminal differentiation subcircuit of the skeletogenic territory is relatively flexible and that existing genetic variation influencing the expression of genes within this subcircuit of the GRN likely allowed for the rapid evolution of skeletal anatomy between species. Comparative network analysis can thus provide a valuable framework to guide future empirical studies that investigate the functional impact of specific regulatory changes on the development of derived traits such the highly modified larval skeleton of *H*. *erythrogramma*.

### Coexpression Analyses Extend the Search for Candidate Genes from the GRN to the Rest of the Transcriptome

The genetic basis for the evolution of lecithotrophy is unlikely to reside only in genes that are part of the GRN. In order to broaden the search for gene expression changes outside the GRN, we explored the feasibility of using coexpression analysis to extend the search to the full transcriptome.

This approach did have limitations, primarily due to the nature of the underlying dataset. Because we were concerned with early development, for practical reasons our data came from whole embryos as opposed to isolated lineages or tissues. This makes it challenging to disentangle the impact of expression changes on developmental processes that occur simultaneously (e.g., skeletal and gut differentiation). Integrating tissue-specific samples and additional time-points will help to alleviate this issue in future investigations.

Despite these limitations, we were able to identify novel candidates for gut and neural development in planktotrophs. We then followed up with studies in *L*. *variegatus* showing that both of these genes are expressed in the predicted region of the developing embryo. These results offer the promise that comparative developmental time course data at the level of the whole transcriptome can provide novel candidate genes for expression changes underlying specific derived phenotypes. Importantly, the comparative coexpression approach modeled here is a valuable tool for gene discovery and can be readily implemented in other systems.

## Materials and Methods

### Sample Collection

*H*. *erythrogramma* and *H*. *tuberculata* adults were collected near Sydney, Australia, and *L*. *variegatus* adults were collected at the Duke University Marine Lab in Beaufort, North Carolina, USA. Larvae were reared following standard protocols [102]. We sampled unfertilized eggs in addition to six developmental stages, replicated in biological triplicate, for each species: 4-cell stage, 16-cell stage, 32-cell stage, mesenchyme blastula, midgastrula, and early larva. For each stage, approximately 300 larvae were placed in RNAlater (Qiagen) and shipped to Duke University for RNA preparation and sequencing.

### RNA Preparation and Sequencing

RNA was extracted for each sample using a Qiagen RNeasy kit. RNA quantity was measured using a NanoDrop, and quality was assessed with a BioAnalyzer. Two micrograms of total RNA were used as input for the Illumina Tru-Seq Library Preparation Kit v2, and libraries were prepared according to the manufacturer’s instructions. Libraries were sequenced on an Illumina Hi-Seq 2000 at Duke’s Sequencing and Genomic Technologies Shared Resource. We sequenced two of the three replicates with 50-bp paired-end sequencing and the third replicate with 50-bp single-end sequencing.

### Transcriptome Assembly and Annotation

For each species, we used Trinity [103] to carry out de novo assembly of a reference transcriptome from the paired-end reads (*L*. *variegatus* assembly: N50 = 2,026, minimum contig length = 201, maximum contig length = 27,428; *H*. *tuberculata* assembly: N50 = 2,360, minimum contig length = 201, maximum contig length = 20,779; *H*. *erythrogramma* assembly: N50 = 2,264, minimum contig length = 201, maximum contig length = 18,959). For the *H*. *erythrogramma* assembly, we included paired-end read samples from our previous work in order to obtain more complete gene models [104]. Following assembly, putative open reading frames (ORFs) were predicted and extracted from each reference transcriptome with OrfPredictor [105]. To reduce redundancy, we used Cap3 to merge highly similar ORFs within each species using default parameters [106]. Our next goal was to generate a consensus set of references, in which ORF models were matched, both in terms of sequence similarity and length, between all three species. To generate consensus alignments between species, we used BLAT with default parameters [107], and only regions that aligned between all three species were retained for further analysis. Although this resulted in the loss of some gene models, the generation of matched references was necessary to make accurate comparisons of transcript abundance across species. Consensus gene models were blasted against the *S*. *purpuratus* peptide database v. 3.1 with an *e*-cutoff of 1e^−10^. Using this approach, we were able to annotate 10,882 unique genes in our matched reference set. With this set, we assigned gene models to the manually curated sea urchin gene ontology outlined by Tu et al. [108], and the number of genes assigned to this ontology are provided in the supplement (S5 Table). GO classifications for *S*. *purpuratus* gene models were obtained using Blast2GO v. 3.1.1 and the NCBI human nonredundant database and were used for categorical enrichment analyses [109].

### Read Mapping and Data Normalization

For each species, quality-filtered RNA-seq reads (left-reads/single-reads) were mapped to the appropriate matched reference assembly with Bowtie, and abundance estimation was performed with RSEM v. 1.2.3 using default parameters [110,111]. Counts were combined for isoforms of the same gene model, and counts per sample prior to normalization are provided in the supplement (S6 Table). The *calcNormFactors* function of the R package edgeR v. 3.10.2 was used to normalize gene counts [112]. Only gene models with more than five counts in at least six samples were kept for further statistical analysis, resulting in 10,713 gene models. Within each species, if a gene’s average expression across the time-course did not exceed five counts per million (cpm), it was designated as “VLE” in further analyses.

### PCA

To assess variance among developmental stages and replicates, we performed a PCA using the R function *prcomp*. Normalized, log_2_-transformed counts from the 10,713 gene models described above were used as input (S1 Data). In a separate analysis, we again used *prcomp* to assess the variance among gene expression profile shapes. Mean expression profiles of normalized, log_2_-transformed counts were standardized prior to analysis using the Mfuzz function *standardise* [113], and genes designated as VLE were excluded (S3 Data). Using the rotated data from this PCA, we calculated scaled distance measures, or “jump scores” between orthologs (*L*. *variegatus—H*. *erythrogramma*) and (*L*. *variegatus—H*. *tuberculata*) with respect to the first two PC loadings: $d=\sqrt{{0.59(x}_{Lv}-\;x_{He}{)}^{2}+\;0.18(y_{Lv}-\;y_{He}{)}^{2}}$ and $d=\sqrt{{0.59(x}_{Lv}-\;x_{Ht}{)}^{2}+\;0.18(y_{Lv}-\;y_{Ht}{)}^{2}}$. For this analysis, the variance explained by PC1 and PC2 was 59% and 18%, respectively. In cases where the expression profile in a given species was VLE, and therefore not included in the PCA, the coordinates were assigned as (0,0).

### Cluster Analysis

For our comparative clustering analysis, we used the R package Mfuzz v. 2.28.0 [113], which employs a fuzzy c-means clustering strategy optimized for the analysis of time-course expression data. Mfuzz offers the following advantages over traditional clustering methods: (1) it is more noise-robust than conventional hard clustering methods (e.g., *k*-means and hierarchical clustering), and (2) it differentiates how closely genes are associated with a given cluster by assigning a membership value based on the similarity of each gene’s expression to the overall pattern of the cluster [114]. Further, Mfuzz is able to calculate membership values for new, independent data based on existing clusters. This aspect of Mfuzz was critical to our comparisons of gene expression profiles within a phylogenetic framework, as it allowed us to generate a background model of expression dynamics in our outgroup *L*. *variegatus*. We then used this model as a baseline to compare expression profiles in *H*. *tuberculata* and *H*. *erythrogramma*.

To implement our comparative clustering strategy, we used the function *mfuzz* to cluster mean expression profiles of log_2_-transformed, normalized gene counts in *L*. *variegatus*. Expression profiles were standardized prior to analysis using the function *standardise*. The number of clusters was set to the largest number for which the correlation between any pair of cluster centroids was less than 0.85, allowing for distinct cluster shapes. Therefore, we set the number of clusters to *c* = 7. Genes previously designated as VLE were not included as input. We used the function *mestimate* to determine the optimal fuzzifier coefficient, which was set to 1.55 (S2 Data). To illustrate the relationship between individual expression profiles to cluster centroids, we used the function *mfuzz*.*plot2* (S7 Fig).

Using the *membership* function, we separately assigned *H*. *tuberculata* and *H*. *erythrogramma* genes to these clusters based on the membership value of individual *H*. *tuberculata* and *H*. *erythrogramma* gene expression profiles to the *L*. *variegatus* cluster centroids. Cluster assignments and associated membership values are provided in the supplement (S1 Table). Overlap matrices of cluster membership were plotted with the *labeledheatmap* function of the R package WGCNA v. 1.47 [115].

### Robustness of Cluster Analysis

To assess the robustness of our comparative clustering strategy, we altered the number of initial clusters to *c* = 5, *c* = 7, *c* = 9 (S8 Fig, S5, S2, S6 Data). We used the Mfuzz functions *overlap* and *overlap*.*plot* with default parameters to examine global cluster structure and the R function *cor* to assess the correlation between individual expression profiles and cluster centroids with increasing *c*. We found that as *c* increased, (1) the amount of overlap between clusters increased (i.e., clusters were less distinct) (S9A Fig), and (2) the correlation between individual expression profiles and cluster centroids also increased (S9B Fig). Importantly, altering the number of initial clusters did not dramatically affect the proportion of genes assigned to conserved, diverged or branch-specific categories at either the whole-transcriptome or network level (S2 Table). Further, the branch-specific increase in cluster jumps in *H*. *erythrogramma* is significant (Fisher’s exact test, adjusted *p* < 0.05) regardless of initial cluster number.

### Categorical Enrichment Analysis

To test for significant enrichment of GO Biological Process categories, we used the *runGSAhyper* function of the R package Piano v. 1.8.2 [116]. As a background list, we used the set of all 10,713 gene models as well as the reduced set of 5,469 genes that exhibited a branch-specific change in expression (i.e., diverged and conserved genes were excluded). FDR corrections for multiple comparisons were calculated using the Benjamini–Hochberg method [117]. This analysis was repeated for *c* = 5 and *c* = 9 clusters (S7 Table).

### GRN Curation

We curated our GRN (Fig 4B) using the well-established sea urchin GRN database (http://sugp.caltech.edu/endomes/). Additional regulatory components for the skeletogenic territory (i.e., *C-lectin*, *Sm37*, *Sm29*, *Sm49*, *p58A*, *p58B*, *p19*, *p16rel1*, *p16rel2*, *p133*, *net7*, *can1*, *pks2*, and *lasp1*) were obtained from Rafiq et al. [11], while additional EM components (i.e., *Apobec*, *Pitx2*, *Six1/2*, *ScratchX*, *FoxC*, *Xlox*, *Cdx*, *Wnt10*) were obtained from Rast et al. [118], Materna et al. [15], and Annunziata and Arnone [18]. For clarity, known GRN components that are not present in our matched assembly are either represented as grey text or are not included in our representation of the network.

### Cloning of Candidate Genes

The full-length coding sequences for candidate genes, *Nkx6-1* and *Mab21L2* were obtained by designing primers against the *L*. *variegatus* transcriptome data set: Nkx6-1-F: 5’- CAACTTGCCGCATTACATAGCATGACGG-3’; Nkx6-1-R: 5’- CAATGTCTGCTTCGACGACCTCGTC-3’; Mab21L12-F: 5’- CCCAGTCTAAACTCCTCTACCAGCTG-3’; Mab21L12-R: 5’- CAGCCTGATCGAGAGCAGCATGAG-3’. Additional reverse primers for each gene containing a 5’ T7 promoter sequence were ordered and used to make in situ hybridization probes.

### Fixation and Whole Mount In Situ Hybridization

*L*. *variegatus* larvae were fixed overnight at 4°C in 4% paraformaldehyde, washed with artificial seawater, and stored in methanol at −20°C. Digoxigenin-11-UTP-labeled RNA probes were synthesized as previously described and used at 1 ng/μL [16]. Hybridization took place at 65°C. Alkaline phosphatase-conjugated anti-DIG antibodies (Roche, 1:1500) were used to visualize probe localization, and NBT/BCIP (Roche) was used to develop the color reaction. For double fluorescent in situ hybridizations, larvae were hybridized with both a DIG-labeled probe and a Fluorescein-12-UTP labeled RNA probe. Expression of both probes was visualized using Horseradish Peroxidase conjugated (anti-DIG and anti-FLU) antibodies (Perkin Elmer), and fluorescence was developed using a Tyramide Signal Amplification system (Perkin Elmer). Larvae were imaged with a Zeiss Axioplan2 upright microscope.

### Data Access

Transcriptome assemblies, sequencing files, and supplementary data files (S1–S6 Data) are available from the Dryad Digital Repository [119].

## Supporting Information

S1 Fig Life history strategy is a relatively minor component of gene expression divergence across the transcriptome. PCA of gene expression (S1 Data). PC2 explains 18% of the variation and separated *L*. *variegatus* from the two *Heliocidaris* species, corresponding to their phylogenetic relationships. PC3 explains 13% of the variation and separated species by life history strategy.
(EPS)

S2 Fig Gene expression divergence is highest in the egg and tends to decrease across development. Comparisons of mean expression divergence (1 - ρ, Spearman’s correlation coefficient) between species for each developmental stage (E, Egg; 4C, 4 cell-stage; 16C, 16 cell-stage; 32C, 32 cell-stage; B, Blastula; G, Gastrula; EL, Early Larva) computed from S1 Data. Error bars represent 95% confidence intervals based on bootstrapping analysis of 10,713 orthologs randomly sampled with replacement 1,000 times.
(EPS)

S3 Fig Jump score distributions. Distribution of jump scores calculated from *L*. *variegatus* to *H*. *tuberculata* (*x*-axis) and from *L*. *variegatus* to *H*. *erythrogramma* (*y*-axis) (S4 Data). Jump scores are colored according to comparative clustering classification: *H*. *erythrogramma* branch jumps (purple), *H*. *tuberculata* branch jumps (blue), genus-level changes (grey), conserved orthologs (dark grey) and diverged orthologs (white) are shown.
(EPS)

S4 Fig Network genes exhibit both conserved and divergent expression profiles between species. Expression profiles for example gene cases involved in **(A)** EM specification, **(B)** endoderm development, **(C)** coelomic pouch development, **(D)** skeletogenic patterning, **(E)** skeletogenic biomineralization, and **(F)** ectoderm development. Biological replicates are represented as circles and average expression profiles across replicates are represented as lines. Expression values below the horizontal line are less than 5 cpm and are designated as VLE (S1 Data).
(EPS)

S5 Fig Generalized categories of GRN change. The majority of *H*. *erythrogramma*-specific branch jumps in the GRN can be further classified according to the following general categories: overall accelerated expression (green), delayed expression (blue), or reduced expression (yellow). Jumps into the VLE group are represented by open boxes, and cases that do not fit into a generalized category are orange.
(EPS)

S6 Fig Coexpression analysis pinpoints changes that may be important for the evolution of lecithotrophic-specific traits in *H*. *erythrogramma*. Expression profiles for example gene cases that are **(A)** expressed in all three species, but accelerated specifically in *H*. *erythrogramma* or that are **(B)** VLE in the planktotrophs but not in *H*. *erythrogramma*. Biological replicates are represented as circles, and average expression profiles across replicates are represented as lines. Expression values below the horizontal line are less than 5 cpm and are designated as VLE (S1 Data).
(EPS)

S7 Fig Fuzzy c-means clustering identifies general patterns of gene expression across development in *L*. *variegatus*. To illustrate major patterns of gene expression, seven clusters detected by fuzzy c-means clustering are shown (S2 Data). The membership score (ms) of a given expression profile within a cluster is represented by color, with red (ms = 1) indicating the highest association.
(EPS)

S8 Fig Comparison of cluster centroids generated with varying cluster number *c*. Cluster centroids identified in the outgroup *L*. *variegatus* are shown for *c* = 5, *c* = 7, and *c* = 9 (S2 Data). The number of genes in each species that were assigned to a given cluster are also shown. Green = *L*. *variegatus*, blue = *H*. *tuberculata*, and purple = *H*. *erythrogramma*. The *x*-axis represents developmental time, and the *y*-axis represents expression change.
(EPS)

S9 FigComparison of global cluster structure with varying cluster number *c*.
**(A)** Overlap plots of global cluster structure for *c* = 5 (red), *c* = 7 (green), and *c* = 9 (blue) obtained by the Mfuzz functions *overlap* and *overlap*.*plot* with default parameters. The overlap between cluster *c* and *l* is defined as: $V_{cl}=\frac{1}{N}\sum_{i=1}^{N}\mu_{ic}\mu_{il}$, where *N* is the total number of gene expression vectors, and μ_*ic*_ and μ_*il*_ are the ms’s of gene *i* to cluster *c* and *l*, respectively. Plots are based on PCA of cluster centroids (circles) and the overlap between individual clusters is represented by grey lines. The line width indicates the strength of the overlap. As *c* increases, overlap increases and cluster structure becomes less distinct. **(B)** Kernel density plots of the correlation between individual expression profiles and cluster centroids in each species for *c* = 5 (red), *c* = 7 (green) and *c* = 9 (blue). As *c* increases, the correlation between individual expression profiles and cluster centroids also increases in each species.(EPS)

S1 Table Cluster assignments and associated ms’s. (XLSX)

S2 Table Cluster number does not dramatically affect the proportion of genes assigned to conserved, diverged or branch-specific categories. (XLSX)

S3 Table Enriched GO categories. (XLSX)

S4 Table GRN component results. (XLSX)

S5 Table Number of gene models assigned to sea urchin ontology categories. (XLSX)

S6 Table Counts per sample prior to normalization. (XLSX)

S7 Table Categorical enrichment results for *c* = 5 and *c* = 9. (XLSX)

## Abbreviations

cpm: counts per million
EM: endomesoderm
eQTL: expression quantitative trait loci
FDR: false discovery rate
GO: gene ontology
GRN: gene regulatory network
ms: membership score
Myr: million years
NSM: nonskeletogenic mesodermal lineage
ORF: open reading frame
PC: principal component
PCA: principal component analysis
VLE: very low expression
