# Correction: Comparative Developmental Transcriptomics Reveals Rewiring of a Highly Conserved Gene Regulatory Network during a Major Life History Switch in the Sea Urchin Genus *Heliocidaris*

**DOI:** 10.1371/journal.pbio.1002489

**Published:** 2016-06-09

**Authors:** The PLOS Biology Staff

## Notice of Republication

This article was republished on March 4^th^, 2016 to correct poor figure quality in the PDF version, which was introduced during the typesetting process. The publisher apologizes for these issues with the figure quality. Please download this article again to view the updated version.

## References

[1] Israel JW, Martik ML, Byrne M, Raff EC, Raff RA, McClay DR, et al (2016) Comparative Developmental Transcriptomics Reveals Rewiring of a Highly Conserved Gene Regulatory Network during a Major Life History Switch in the Sea Urchin Genus *Heliocidaris*. PLoS Biol 14(3): e1002391 doi: 10.1371/journal.pbio.1002391 26943850 10.1371/journal.pbio.1002391 PMC4778923

